# Semi-supervised generative and discriminative adversarial learning for motor imagery-based brain–computer interface

**DOI:** 10.1038/s41598-022-08490-9

**Published:** 2022-03-17

**Authors:** Wonjun Ko, Eunjin Jeon, Jee Seok Yoon, Heung-Il Suk

**Affiliations:** 1 Department of Brain and Cognitive Engineering, Korea University, Seoul, 02841 Republic of Korea; 2 Department of Artificial Intelligence, Korea University, Seoul, 02841 Republic of Korea

**Keywords:** Rehabilitation, Learning algorithms

## Abstract

Convolutional neural networks (CNNs), which can recognize structural/configuration patterns in data with different architectures, have been studied for feature extraction. However, challenges remain regarding leveraging advanced deep learning methods in BCIs. We focus on problems of small-sized training samples and interpretability of the learned parameters and leverages a semi-supervised generative and discriminative learning framework that effectively utilizes synthesized samples with real samples to discover class-discriminative features. Our framework learns the distributional characteristics of EEG signals in an embedding space using a generative model. By using artificially generated and real EEG signals, our framework finds class-discriminative spatio-temporal feature representations that help to correctly discriminate input EEG signals. It is noteworthy that the framework facilitates the exploitation of real, unlabeled samples to better uncover the underlying patterns inherent in a user’s EEG signals. To validate our framework, we conducted experiments comparing our method with conventional linear models by utilizing variants of three existing CNN architectures as generator networks and measuring the performance on three public datasets. Our framework exhibited statistically significant improvements over the competing methods. We investigated the learned network via activation pattern maps and visualized generated artificial samples to empirically justify the stability and neurophysiological plausibility of our model.

## Introduction

Brain–computer interfaces (BCIs) provide communication pathways between an enhanced or wired brain and an external device (e.g., robotic arm, exoskeleton, electric wheelchair) by measuring brain activities. Because of their practicality, non-invasive electroencephalogram (EEG)-based BCI systems are widely used^1^. Earlier, Zander et al. categorized user-centered BCIs into active/reactive and passive^2^, which are both used for directed control and accessing or interpreting changes in the user’s brain state, respectively. Our focus in this paper, is on the active BCI. In active and reactive BCIs, two types of brain signals such as *evoked* and *spontaneous*, depending on approaches of inducing brain signals, are mostly considered. Evoked BCIs take advantage of unintentional electrical potentials reacting to external or internal stimuli. Examples include P300, steady-state visually evoked potentials (SSVEPs), and steady-state somatosensory evoked potentials. These are called evoked potentials. On the other hand, spontaneous BCIs involve internal cognitive processes such as event-related (de)synchronization (ERD/ERS) in sensorimotor rhythms, induced by the imagination of movements, or motor imagery (MI), and physical movements.

Thanks to voluntary-induction, an MI-based BCI implies great values in both clinical and application domains^3^. Based on prior neurophysiological knowledge, MI-based BCI systems undergo spatio-spectral-temporal filtering to extract features (e.g., a common spatial pattern^3^ or its variants^4,5^). These methods, however, mostly determine class-discriminative feature representations independently from the following classifier training stage. Meanwhile, deep learning has achieved great success in discovering feature representations, jointly learned with a classifier in an end-to-end manner, across various applications^6,7^. In particular, the convolutional neural network (CNN) aids in maintaining the structural or configurational information in the data during training among various deep learning methods. In this respect, developing a novel CNN architecture for representation learning has taken center-state in the BCI community as well^7–14^.

**Figure 1:**
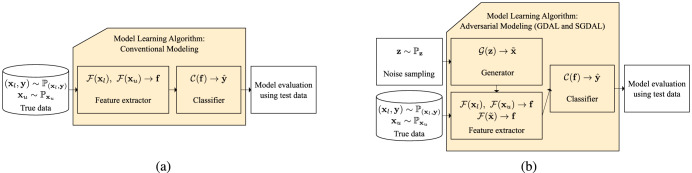
Comparison of learning schemes. (**a**) A conventional non-adversarial learning uses a training dataset (labeled and/or unlabeled) to learn the machine learning algorithm. On the other hand, (**b**) adversarial learning leverages artificially generated samples from random noise to learn the algorithm.

However, developing a CNN-based feature extractor and classifier for BCIs is still challenging, mainly for two reasons. First, deep learning is a data-hungry method whereas normal BCI systems acquire, in general, a limited number of training samples, i.e., less than hundreds during a calibration session^15^. This is time-consuming and hinders its practical applicability. In smaller-sized datasets, *transfer learning*^16^ has been considered a remedy via the exploitation of samples from multiple subjects jointly, thus constructing a larger dataset. Because of the significantly high variabilities in EEG signals among subjects or sessions, and unpredictable artifacts, however, the performance improvements reported in the literature were limited^17,18^. Second, even though a CNN can identify complex patterns latent in a dataset, interpreting learned model parameters (patterns) in a neurophysiological viewpoint remains complicated.

In this work, we propose a novel deep semi-supervised generative and discriminative adversarial learning framework for BCI that generates artificial samples to boost the generalization power of a model. It should be noted that, taking inspiration from Odena’s work on semi-supervised adversarial learning, we have devised the training strategies by leveraging recent advanced techniques, particularly, to stabilize generator training, such that our method fits EEG representation and classification learning. It is comparable to previous works on transfer-learning based approaches that have mostly used real samples^16,17^. Specifically, the proposed framework is designed for two reasons: (1) learning and representing EEG signals on the latent space in the viewpoint of generative models^19,20^; (2) synthesizing artificial samples with indistinguishable signal patterns from those of the real samples of a target subject. Thereby, the proposed framework allows us to possibly learn more general feature representations from the artificial samples, thus enhancing classification accuracies. In essence, this work is inspired by Lotte’s work^21^, which demonstrated the use and effects of artificial EEG samples in constructing a BCI system.

In terms of explaining a model’s prediction utility for interpreting learned model parameters, Binder et al. proposed a layer-wise relevance propagation (LRP) method, which can generally be applied to deep feedforward networks for the explanation of output decision-making^22^. Meanwhile, because of the notorious difficulty encountered when interpreting the learned parameters of a CNN with a trained model analysis, most existing deep-learning based methods^7^ focus less on the neurophysiological interpretation of learned parameters. Schirrmeister et al.^8^ and Lawhern et al.^10^ visualized the learned spatial filters and calculated the summary statistics of pairwise correlations in inputs, unit activation values, and outputs, in their independent works. Sturm et al.^23^ applied the layer-wise relevance propagation (LRP)^22^ to determine which input values contribute to the final output either positively or negatively in terms of relevance, estimated via a backpropagation-like method to BCI studies and visualized decision-explanation in topographic maps^23^. In this work, we conducted experiments using an existing CNN architecture in the literature and investigated the learned models in terms of an activation pattern map^24^, which is better suited for interpreting and understanding the learned weights topologically. By regarding the EEG classification as a backward problem, i.e., estimating the source signal of a user’s intention from EEG observations, we transformed the learned weights into a forward formulation and represented those trained weights in the form of activation pattern maps^24^, with which we could investigate and interpret the neurophysiological plausibility of the learned spatial weight parameters.

**Figure 2:**
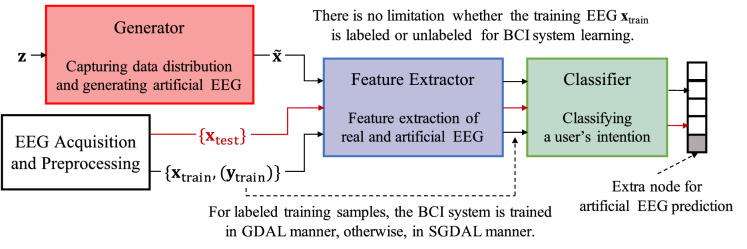
A schematic illustration of the proposed semi-supervised generative and discriminative deep adversarial learning framework for MI-based BCI. The black and red arrows denote data or features flows during a training step and a test step, respectively. (GDAL: generative and discriminative adversarial learning, SGDAL: semi-supervised GDAL).

The main contributions of our work are as follows:

- First, we propose an adversarial modeling framework for MI-based BCI in both supervised and semi-supervised manners. More precisely, in our work, we focus mainly on applying various methodological findings in generative adversarial learning to deep learning-based BCI thereby tackling one of the most important problems in deep learning-based BCI, namely boosting generalization with a limited number of training samples.
- Second, the proposed method achieved reasonably high accuracy with limited training samples on over three public datasets, and exhibited statistical significance compared to the competing methods considered in our experiments.
- Last, we introduce an approach to analyze the learned network parameters by transforming them into activation patterns and illustrating them topographically for visual inspection and neurophysiological investigation.

This is an extended version of our previous work^25^. We, specifically conducted more exhaustive experiments by further exploiting other deep network architectures, namely Shallow ConvNet and Deep ConvNet^8^, and performing experiments over two other public datasets. It should also be noted that we repeated the experiments ten times with various scenarios for more robust and conclusive results. Last, we also analyzed the proposed method from a neurophysiological perspective via activation pattern maps^24^.

## Related work

Learning class-discriminative spatio-temporal features of EEG data remains challenging in both theory and practice. Although numerous prior studies using different forms of brain signals have been conducted, in the present study, we focus on MI-based BCIs. In addition, we briefly introduce the concept of generative adversarial networks (GANs) because of its relevance to our framework.

### EEG-based MI classification

Many studies developed decoding models of EEG data, for which machine learning has played pivotal roles over the past decades. A conventional, i.e., non-adversarial, *(semi-)supervised learning* framework is generally composed of two parts: a feature extractor ${\mathcal {F}}(\cdot )$ and a classifier ${\mathcal {C}}(\cdot )$, as shown in Fig.,1a. From the training data (and the corresponding label, if available,) the feature extractor attempts to learn the distribution ${\mathbb {P}}_{({\mathbf {x}}_l,{\mathbf {y}})}$ and/or ${\mathbb {P}}_{{\mathbf {x}}_u}$, where subscripts *l* and *u* denote labeled and unlabeled, respectively, and extracts feature ${\mathbf {f}}$ for classification. A classifier then outputs a corresponding label ${\hat{\mathbf {y}}}$ from the feature ${\mathbf {f}}$.

Edelman et al.^1^ used principal component analysis to classify complex motor imagery EEG tasks, whereas Blankertz et al.^3^ and Ang et al.^4^ both used a spatial filtering based method, i.e., common spatial pattern (CSP), for MI-based BCI. Suk and Lee^26^ decoded MI-EEG by jointly optimizing spatio-spectral filters in a Bayesian framework. Meanwhile, Meng et al.^27^ also classified EEG by using optimized spatio-spectral features based on mutual information. Further, Xie et al.^28^ also discriminated MI-EEG data, using a tangent space of the sub-manifold algorithm by extracting a Riemannian sub-manifold and performing classification using a support vector machine.

Although existing research mostly focused on MI decoding in a *supervised* manner, Meng et al.^15^ and Li and Guan^29^ independently studied MI-based BCI in a *semi-supervised* manner. In particular, Meng et al.^15^ initialized and trained a weak classifier by using a small-sized training dataset, and finally trained a strong classifier with an iterative procedure by using some portions of a test dataset for label prediction.

Recently, deep learning-based EEG decoding has changed these conventional approaches by combining feature extraction or representation with classifier learning in a unified framework. Specifically, studies have focused on the properties of CNN that efficiently exploit structural or configurational information in feature extraction from EEG data. For example, Schirrmeister et al.^8^ and Fahimi et al.^30^ introduced CNN architectures for raw MI-EEG decoding. Sakhavi et al.^7^ also proposed a training strategy to learn temporal information from MI-EEG signals by using CNN. They modified filter-bank CSP (FBCSP)^4^ to extract temporal features and selected discriminative features with a mutual information-based method. Further, Lawhern et al.^10^ effectively exploited subtypes of convolution, the depthwise and the separable convolution, thereby dramatically reducing the number of tunable parameters of a deep CNN used in their work. Finally, Ko et al.^14^ also exploited the separable convolution with multi-scale feature extraction architecture, represented EEG signals. A CNN that takes these selected features as input subsequently learns more complex representations. In their analysis, they visualized the temporal kernels of the CNN. However, despite being an interesting analysis from a model selection perspective, this approach used hand-crafted features as network inputs and was unable to provide any neurophysiological insights.

Deep learning-based BCI can potentially enhance classification accuracy, thus advancing their practical applicability. However, it still suffers from very fundamental requirements for a large number of training samples and an inability to interpret or understand the learned model.

Unlike aforementioned traditional approaches, in this work, we exploit an adversarial modeling by introducing another neural network, a generator ${\mathcal {G}}(\cdot )$, as shown in Fig. 1b.

### Generative adversarial networks

In the deep-learning community, Goodfellow et al.^19^ proposed an innovative learning paradigm with GANs for data generation or augmentation. Original GANs comprised two neural networks, namely a generator and a discriminator. A generator is trained to produce an artificial sample by mapping a random noise to a realistic sample, whereas a discriminator learns to distinguish real data from artificially generated data^31^. GANs have become immensely popular in various fields and applications such as image generation^32,33^, audio synthesis^34^, super-resolution^35^, classification or regression^36,37^, and domain adaptation^38^ tasks.

The (generative) adversarial learning has also been applied to BCI tasks for well generalization^39^. For instance, Tan et al.^40^ converted raw EEG signals to EEG optical flow images and obtained a general feature extractor for EEG optical flow images and ImageNet by adversarial learning to build a classification network capable of classifying category labels. Özdenizci et al.^41^ built an adversarial deep learning method to identify a person using EEG signals as biometrics. Additionally, Özdenizci et al.^42^ also applied an adversarial learning concept to reduce the inter-subject variability^16^.

In the viewpoint of EEG data augmentation, Roy et al.^43^ and Krishna et al.^44^ exploited the original version of GANs^19^. Pascual et al.^45^ used conditional LSGAN^46^ concept. Further, Zhang et al.^47^ and Zhang and Liu^48^ both used DCGANs^33^ for motor imagery EEG signal generation. Fahimi et al.^49^, Aznan et al.^50^ and Lee et al.^51^ also used DCGANs for synthesizing realistic EEG signals. Hartmann et al.^52^ and Ko et al.^25^ both exploited Wasserstein GANs with a gradient penalty^53^ to generate artificial EEG samples. Moreover, Panwar et al.^54^ and Lu et al.^55^ both exploited Wasserstein GANs with a gradient penaly and a condition vector for generating artificial EEG signals. Furthermore, Corley and Yufei^56^ upsampled spatial resolution of EEG using GANs^19^. In particular, they organized Wasserstein GANs^57^ to stabilize the training procedure, however, their work was based on developing a generative model in an unsupervised manner, while our proposed work focuses on establishing a well-stabilized discriminative model in a semi-supervised manner. Moreover, Wei et al.^58^ conducted multi-source domain adversarial domain adaptation to reduce the rapid serial visual presentation data acquisition phase. In our work, we also focus on the reducing calibration efforts of an MI-based BCI in the semi-supervised manner. Finally, Fahimi et al.^49^ implemented Wasserstein GANs^57^ to synthesize SSVEP samples, thereby augmenting the training dataset size.

As the original GANs^19^ are designed to train in an unsupervised manner, they are not necessarily useful for classification tasks, especially in BCIs^41,50,56^. In this regard, inspired by Odena’s work^59^ that extended the original GANs framework by including both a generative model and a classifier simultaneously and presented its validity for classification tasks, we propose a semi-supervised deep adversarial learning framework in this study. More specifically, given CNN architectures proposed for EEG analysis, we propose a strategy to design the structure of a generator network based on the given feature extractor network. Then, we used the feature extraction network and the classifier network as a discriminator in our framework. By doing so, the proposed method effectively exploits an adversarial learning scheme and class-discriminative feature representations for MI-based BCI with a limited number of training samples.

## Experiments and analysis

In this section, we describe datasets used for performance evaluation, our experimental settings, base CNN architectures used for a generator ${\mathcal {G}}$ and a combined feature extractor and classifier, ${\mathcal {F}}\circ {\mathcal {C}}$. Furthermore, we present the classification accuracies of our method and those of competing methods.

### Dataset & preprocessing

We used three BCI Competition datasets, III-3a, III-4a, and IV-2a that consisted of different motor imagery tasks. Importantly, as these datasets have separate training and test trials, we subsequently conducted five-fold nested cross-validation with training samples only for model selection.

- III-3a: This dataset consisted of four motor imagery tasks: left hand, right hand, feet, and tongue. All EEG signals were acquired from three subjects and recorded using 60 Ag/AgCl electrode channels according to a 10-20 system. In addition, the signals were band-passfiltered between 1 and 50Hz, and the sampling frequency was 250Hz.
- III-4a: This dataset consisted of two motor imagery tasks: right hand and foot. All EEG signals were acquired from five subjects, recorded using 118 Ag/AgCl electrode channels according to the 10-20 system, sampled at 1000Hz, and band-pass filtered between 0.05 and 200Hz.
- IV-2a: This dataset consisted of four motor imagery tasks similar to III-3a. All EEG signals were acquired from nine subjects, recorded using 22 Ag/AgCl electrode channels according to the 10-20 system, sampled at 250Hz, and band-pass filtered between 0.5 and 100 Hz.

To have consistently model our deep networks, we first selected 22 channels from III-3a and III-4a and downsampled III-4a to 250Hz to match the sampling of IV-2a. Finally, all datasets were band-pass filtered between 1 and 50Hz. Similar to previous studies^12,25^, we preprocessed the signals by applying a *large Laplacian filter*. Note that when a target channel does not have four nearest neighbor, we just used available channels and their average value to filter the target channel. We then segmented signals of 1 sec in length before the cue to determine baseline signaling. We subtracted the mean value of the baseline from each trial for baseline correction^12,26^. Further, we normalized EEG trials for each subject in a channel-wise manner. That is, we estimated the mean and standard deviation values for each channel independent of all other training samples of a subject and transformed EEG trials to have a zero mean and unit variance by subtracting the mean and dividing with a standard deviation. As for the test samples, we applied the same mean and standard deviation values for normalization. Note that as the multi-channel EEG signals were only shifted and scaled by their respective channel-wise mean and standard deviation values, it reserved inter-channel relations inherent in data. Finally, we removed the first and the last signals of 0.6 sec in length, i.e., ${\mathbf {x}}_l, {\mathbf {x}}_u\in {\mathbb {R}}^{22\times 700}$.

### Experimental settings

Owing to a lack of training samples (only dozens$\sim$hundreds of trials were collected, in total), we were precluded from training the existing deep CNN models without suffering from overfitting. As a remedy, we used a data augmentation strategy involving a sliding window-based voting method. Specifically, we set the size of a window to be approximately 2 secs in length similar to previous studies^8,12,25^ and an exponential power of 2 for efficient GPU computation^60^ (512 time points). Then, we slid it using a stride length of one time point. In our experiments, we used three public datasets of BCI Competition III-3A, 4A, and IV-2A. Basically, each of the datasets was already split into train and test sets for fair evaluation purposes over different methods. Thus, there was no need to consider train/test splitting, and it was guaranteed that no test samples were involved in any of the training steps. Meanwhile, the use of over-segmented samples with a sliding window was to boost the number of training samples for robust network training. By doing so, we produced 189 segments with a sliding window of 22 channels $\times$ 512 time points for a single EEG trial of 22 channels $\times$ 700 time points, i.e., $189=700-512+1$. Then, we fed these segments into our network to make 189 outputs, one for each segment, and made a single decision by means of a majority voting. Under our GPU acceleration setup (NVIDIA Titan RTX), this ensemble strategy was computed within 0.3 sec. This process was carefully performed such that it did not entangle training and test samples for model learning. Further, as the size of a sliding window determines the input dimension of a CNN, to make a determination using only one label for a single test trial, we applied a *voting* strategy^61,62^ with the outputs from all windowed signals of a single trial.

To validate the efficacy of our method, we performed the experiments using two different scenarios.

- Scenario I: It was designed to demonstrate the validity of GDAL by presenting the performance improvements that varied according to the number of training samples. We built two CNN-based models, i.e., one with adversarial learning and one without adversarial learning. We then randomly selected 100, 75, 50, 25, and 12.5% of the training samples for each class and used these samples for training.
- Scenario II: In this scenario, we focused on the use of unlabeled samples for semi-supervised learning, i.e., SGDAL. We randomly selected 75, 50, 25, and 12.5% of the training samples for each class and discarded their labels during training. We compared the results of this scenario to the baseline results of Scenario I.

While training our proposed framework, we set a mini-batch size of 64 within 100 total epochs, an exponentially decreasing learning rate (inital value: $3.0\times 10^{-2}$, decreasing ratio per epoch: $1.0\times 10^{-3}$) and used an Adam optimizer. Note that the proposed framework is adaptable to many kinds of CNN architectures, varying from existing networks in the BCI literature to newly designed ones. In this work, for the feature extractor and the classifier ${\mathcal {C}}\circ {\mathcal {F}}(\cdot )$, we exploited existing CNN architectures^8,12^ as reported in the next subsection. For the generator network ${\mathcal {G}}(\cdot )$, we built a new deep deconvolution network in the reverse order of the feature extractor ${\mathcal {F}}(\cdot )$. Thanks to DCGANs modeling strategy^33^, we removed pooling layers and replaced all nonlinear activation functions to ReLU activation. Finally, for the noise vector ${\mathbf {z}}$ inputted to the generator, we sampled a 100-dimensional vector from Gaussian distribution ${\mathcal {N}}({\varvec{0}}, {\varvec{1}})$.

### Base CNNs for adversarial modeling

For all experiments, we considered three existing CNN architectures: RSTNN^12^, Deep ConvNet^8^, and Shallow ConvNet^8^. Note that these architectures have various forms, e.g., very deep and recurrently repeated convolutions (13 convolutions)^12^, a less deep network with 5 convolutions^8^ and a shallow model with 2 convolutions^8^. Here, we describe the characteristics of each network in brief. For the complete specifications of the architectures, refer to the original paper cited below. All codes used in our experiments are available at ‘http://deepbci.korea.ac.kr/opensource/opensw/.’

**Figure 3:**
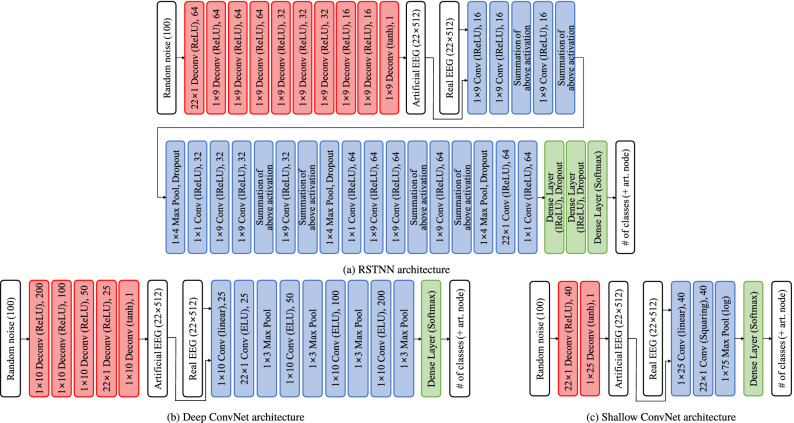
Illustration of three different GAN architectures used in our experiments. In all feature extractors (blue square) ${\mathcal {F}}$, every convolutional layer was activated by leaky ReLU (lReLU), and the corresponding classifiers (green square) ${\mathcal {C}}$ were activated by $\tanh$. In these combined feature extractors and classifiers, real input EEG and artificially generated EEG that have a matrix form of 22$\times$512 were inputted and predictions of networks were outputted (artf. node denotes a node for artificially generated EEG). Meanwhile, in all generators (red square) ${\mathcal {G}}$, every deconvolutional layer was activated by ReLU with the except of the final layer activated by $\tanh$. In these generators, a random noise vector ${\mathbf {z}}\in {\mathbb {R}}^{100}$ was inputted, and an artificially generated EEG (22$\times$512) was outputted.

- RSTNN^12^ is inspired by RCNN^63^, which achieved promising results in the motor execution of EEG decoding. This network consists of a number of recurrent convolutional layers. A recurrent convolutional module in RSTNN^12^ is composed of three recurrent convolution layers (temporal convolutional kernels, 1$\times$9) and spatial features based on spatial convolution layers (spatial convolutional kernel, the number of channel$\times$1). Following the feature extraction, fully connected layers are used to classify the features. To build a generator ${\mathcal {G}}$, we assembled deconvolutional layers in the reverse order of the feature extractor and with ReLU activation, with the exception of the output layer, for which we used a $\tanh$ activation function. Originally, RSTNN^12^ has three *spatio-temporal* modules, thus we retained the original settings for III-3a and IV-2a, however, we used a single module for III-4a because of significantly smaller number of training trials. See Fig.,3a for the detail structure.
- A Deep ConvNet^8^ consists of a temporal convolution layer followed by a linear activation, a spatial convolution layer with an ELU activation, three temporal convolution layers, each of which is applied with an ELU activation, and an output layer with a softmax mapping function. See Fig. 3b for the used architecture.
- A Shallow ConvNet^8^ is designed with a pipeline of one temporal convolution layer with a linear activation, spatial convolution layer with a squaring activation, and an output layer with a softmax mapping function for prediction. See Fig. 3c for more details.

**Table 1:** Performance evaluation for every case. Ratio rows denote number of used training samples of each dataset, which is shown in the Dataset rows. Baselines column shows the performance of a conventional CSP and LDA method (CSPwLDA column), Lotte’s artificial EEG generation using time-frequency domain EEG^21^ (ADG), and Lotte’s semi-supervised CSP and LDA method^21^ (SS-ADG). Shallow ConvNet, Deep ConvNet^8^, and RSTNN^12^ columns indicate the CNN architecture to demonstrate the classification performance of conventional modeling (Vanilla), the proposed adversarial modeling (GDAL), and semi-supervised adversarial modeling (SGDAL). * and ** denote $p<0.05$ and $p<0.005$, respectively.

		Baselines			Shallow ConvNet^8^			Deep ConvNet^8^			RSTNN^12^		
Ratio	Dataset	CSPwLDA	ADG^21^	SS-ADG^21^	Vanilla	GDAL	SGDAL	Vanilla	GDAL	SGDAL	Vanilla	GDAL	SGDAL
100%	III-3a	.75 ± .12	N/A	N/A	.75 ± .12	.77 ± .11**	N/A	.77 ± .10	.78 ± .10**	N/A	.74 ± .11	.76 ± .12	N/A
	III-4a	.80 ± .14	N/A	N/A	.89 ± .06	.89 ± .05	N/A	.87 ± .06	.88 ± .06	N/A	.75 ± .10	.77 ± 10	N/A
	IV-2a	.62 ± .15	N/A	N/A	.67 ± .14	.68 ± .14*	N/A	.66 ± .14	.68 ± .14**	N/A	.66 ± .14	.67 ± .14*	N/A
75%	III-3a	.74 ± .12	.73 ± .11	.69 ± .13	.71 ± .12	.72 ± .12*	.74 ± .12**	.77 ± .10	.77 ± .10	.79 ± .09*	.73 ± .12	.74 ± .13*	.75 ± .13**
	III-4a	.76 ± .14	.75 ± .15	.63 ± .15	.74 ± .10	.76 ± .09*	.77 ± .09*	.77 ± .09	.76 ± .09	.76 ± .09	.71 ± .08	.72 ± .07	.73 ± .07*
	IV-2a	.58 ± .13	.56 ± .13	.53 ± .16	.63 ± .14	.65 ± .14*	.66 ± .14**	.64 ± .14	.65 ± .14*	.66 ± .13*	.63 ± .13	.65 ± .13*	.66 ± .13*
50%	III-3a	.67 ± .15	.65 ± .16	.64 ± .16	.68 ± .13	.69 ± .12*	.70 ± .12*	.71 ± .11	.73 ± .10*	.74 ± .09*	.70 ± .12	.71 ± .12	.71 ± .09*
	III-4a	.72 ± .15	.71 ± .16	.62 ± .16	.72 ± .07	.74 ± .09**	.74 ± .09*	.71 ± .10	.71 ± .09	.72 ± .11	.70 ± .08	.71 ± .08*	.72 ± .08*
	IV-2a	.56 ± .14	.56 ± .14	.51 ± .16	.61 ± .13	.61 ± .13	.62 ± .13	.60 ± .13	.61 ± .13*	.62 ± .13*	.60 ± .13	.61 ± .13*	.62 ± .13*
25%	III-3a	.62 ± .20	.60 ± .20	.63 ± .15	.66 ± .13	.68 ± .12**	.69 ± .12*	.68 ± .15	.70 ± .13**	.71 ± .14*	.68 ± .11	.69 ± .14*	.71 ± .11**
	III-4a	.72 ± .15	.72 ± .14	.61 ± .15	.72 ± .09	.74 ± .09*	.75 ± .09*	.72 ± .09	.73 ± .10**	.73 ± .09*	.68 ± .08	.69 ± .08*	.70 ± .07*
	IV-2a	.53 ± .14	.53 ± .13	.49 ± .17	.54 ± .13	.56 ± .13**	.56 ± .13*	.54 ± .13	.55 ± .12*	.55 ± .12**	.54 ± .13	.55 ± .13*	.56 ± .13*
12.5%	III-3a	.59 ± .20	.57 ± .19	.50 ± .20	.65 ± .10	.66 ± .11*	.68 ± .11*	.66 ± .16	.68 ± .16*	.67 ± .16**	.66 ± .14	.66 ± .14	.66 ± .12
	III-4a	.65 ± .19	.65 ± .18	.59 ± .16	.67 ± .10	.69 ± .10*	.68 ± .21	.64 ± .10	.65 ± .11*	.66 ± .10**	.63 ± .06	.63 ± .05	.64 ± .04*
	IV-2a	.50 ± .14	.49 ± .14	.45 ± .15	.48 ± .11	.49 ± .11	.50 ± .11*	.48 ± .12	.49 ± .11**	.50 ± .11**	.48 ± .11	.49 ± .11**	.49 ± .11*

### Performance comparison

The experimental results are summarized in Table 1. For comparison with linear models, we built a CSP with LDA (CSPwLDA)^3^ and implemented Lotte’s artificial data generation (ADG) method and Lotte’s semi-supervised CSP (SS-ADG) method^21^. For the linear models, we used 6 filters and regularized covariance for CSP and artificial EEG generation in the time-frequency domain^21^ for ADG. For the ADG method^21^, we used the same settings for CSP and LDA, and generated the same number of artificial samples as that of the removed training samples, i.e., for instance, when ADG has 75% of training samples, it generates 25% for additional training samples. For the SS-ADG method^21^, we also used the same settings for CSPwLDA. Further, we unlabeled the same number of training samples as that of the used ratios. For example, SS-ADG has 75% of labeled training samples and 25% of unlabeled training samples in the case of a 75% ratio.

Even though previous studies have decoded MI-EEG in a *semi-supervised* manner^15,29^, these studies have used unlabeled test data for their methods. Therefore, it is not fair to directly compare these methods with our proposed method. Thus, we did not compare the proposed method with these previous methods^15,29^.

To evaluate and compare the performance among comparative methods, we repeated all experimental scenarios 10 times over three different datasets for more robust results and better generalized conclusions. We also estimated *p*-values to indicate statistical significance between conventional modeling, i.e., ‘Vanilla’ and each (semi-supervised) adversarial modeling i.e., ‘GDAL’ and ‘SGDAL’. With regard to the statistical test, we used the two-tailed Wilcoxon’s signed rank test between a vanilla model and its counterpart GDAL or SGDAL model based on their repeated measures test across 9 subjects’ accuracies. Furthermore, to avoid the *multiple comparison problem*, we adjusted our acquired *p*-values using the Bonferroni’s correction technique. For example, in the comparison between ‘Vanilla’ and its counterpart GDAL scenario, we first estimated *p*-values, then multiplied 45 to adjust *family-wise error rate*.

**Figure 4:**
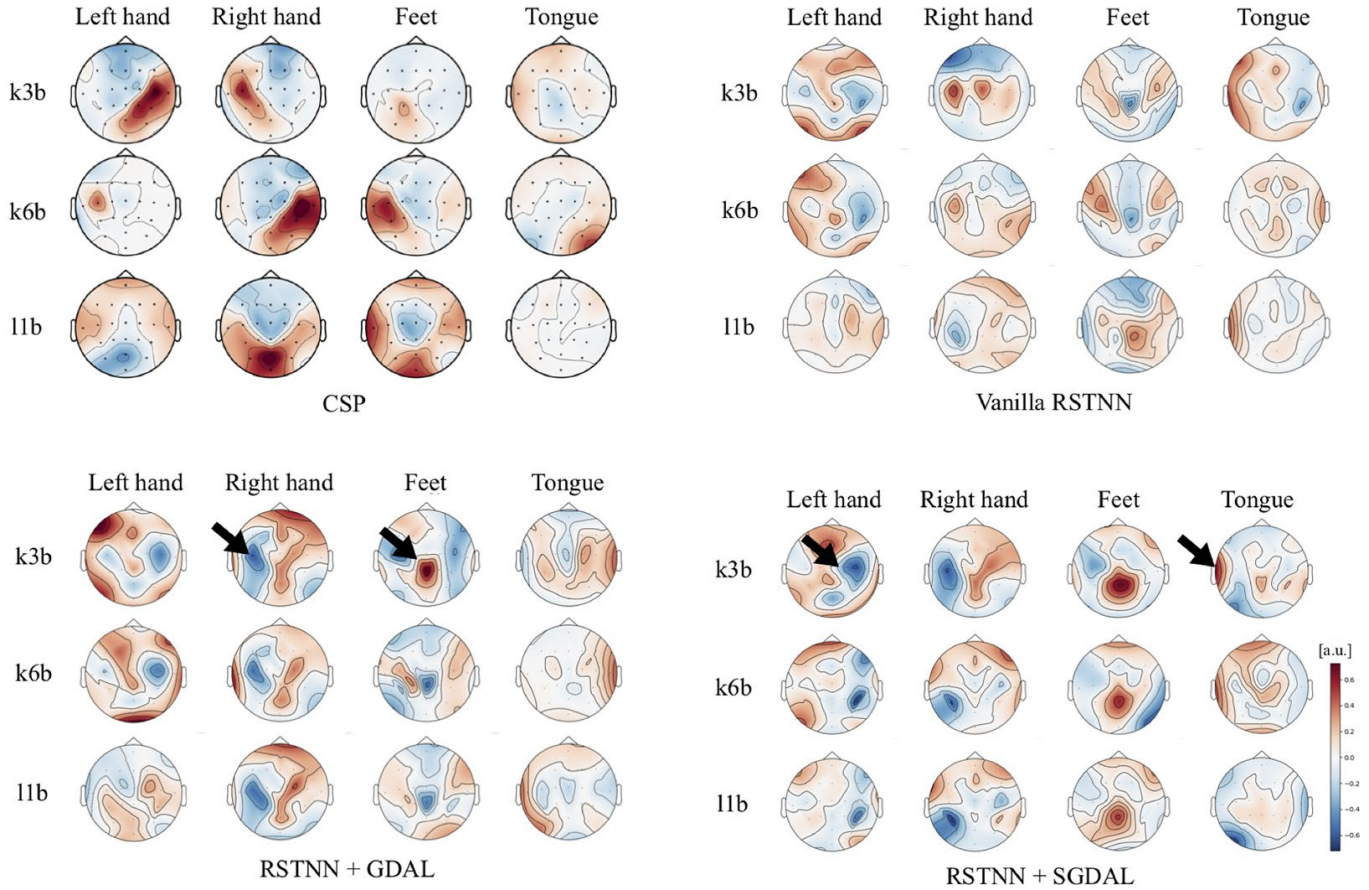
Activation pattern maps for the learned spatial filters in CSP (left top), the learned spatial kernel weights in RSTNN^12^ (right top), the learned spatial kernel weights in RSTNN + GDAL (left bottom), and the learned spatial kernel weights in RSTNN + SGDAL (right bottom). Each column is related to a class, i.e., left-hand, right-hand, feet, and tongue, and each row denotes a subject index.

#### Scenario I

The results of different base CNNs used in this scenario are shown in Table 1. We observed clear improvements in the classification performance in all base CNNs, yielding small *p*-values which indicate a high statistical significance. A noteworthy aspect of this scenario is the relatively large improvement seen in subject 2, 4, 5, and 6 from the IV-2a dataset that was previously regarded as a BCI illiterate, a user who has significant difficulty in using BCI systems^4,28^. Additionally, Deep ConvNet and Shallow ConvNet^8^ exhibited performance improvements when the networks were trained using our GDAL framework. Based on the summary of the accuracies listed in Table 1, it is noteworthy that SGDAL clearly outperformed its counterpart GDAL, whose results were mostly superior to the corresponding Vanilla and Baseline models in all scenarios and datasets, except for the three cases with 75% and 12.5% of dataset III-4a and 12.5% of dataset III-3a. Furthermore, on comparing the three networks, there was no evident trend indicating whether one network was superior to others. However, Shallow ConvNet, which is characterized as a relatively smaller network than Deep ConvNet and RSTNN in terms of learnable parameters, still achieved the highest accuracy in many scenarios. A possible reason for a small network exhibiting better performance than deeper networks could be the limited number of training samples. Nonetheless, as other deep models, i.e., Deep ConvNet and RSTNN, also presented reasonably high performances, they still deserve good candidate networks as a module in our proposed framework. Notably, in every dataset and case, the average performance across subjects with existing CNNs^8,12^ and GDAL was higher than the performance of those with conventionally learned (vanilla) CNNs.

#### Scenario II

As shown in Table 1, the use of the proposed SGDAL led to clear performance improvements with respect to all base CNNs considered in this study. It is noteworthy that the resulting *p*-values were generally less than 0.05, denoting a high statistical significance. We also observed that training with the unlabeled data by using the proposed method improved the performance of the networks in most of the cases.

In every case, the SGDAL method exhibited a higher performance than the vanilla CNNs. Thus, based on these promising results, we conclude that the SGDAL framework proposed in this study can be applicable to *incremental* learning. After training our SGDAL-based BCI system with a few labeled training samples, it was possible to update this system with new test trials involving an unknown label (e.g., unlabeled trials). From the perspective of developing systems, this can be considered as lifelong learning via dynamically self-updating network parameters, which will be our forthcoming research topic.

More importantly, when we used smaller amounts of training samples, i.e., 75, 50, 25, 12.5% of the training samples, Deep ConvNet and Shallow ConvNet^8^ with adversarial modeling, i.e., GDAL or SGDAL showed the highest performance in many cases. Based on these results, we concluded that the proposed method functioned, even with a small training dataset. Thus, we believe that the proposed method has significant potential for applications in situations wherein it is difficult to collect many training samples.

**Figure 5:**
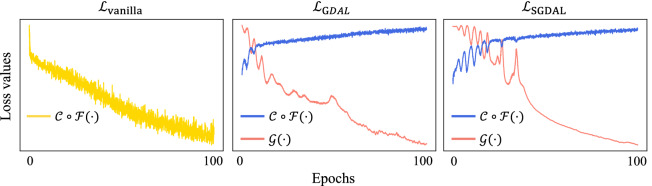
Loss curves of the generator network ${\mathcal {G}}(\cdot )$ and the feature extraction with the classifier network ${\mathcal {C}}\circ {\mathcal {F}}(\cdot )$ for the vanilla training ${\mathcal {L}}_\text {vanilla}$, the generative-discriminative adversarial learning (GDAL) ${\mathcal {L}}_\text {GDAL}$, and the semi-supervised GDAL ${\mathcal {L}}_\text {SGDAL}$.

We plotted loss curves of the generator ${\mathcal {G}}(\cdot )$ in our GDAL and SGDAL framework, and loss curves of the feature extractor and the classifier, i.e., the discriminator ${\mathcal {C}}\circ {\mathcal {F}}(\cdot )$ in our framework and the vanilla training setting in Fig. 5. To be specific, we visualized training curves of Deep ConvNet^8^ trained with 75% of Subject 1’s EEG samples in BCI Competition IV-2a dataset. We observed that the feature extractor and the classifier networks, i.e., ${\mathcal {C}}\circ {\mathcal {F}}$ oscillates at the beginning of training, but gradually stabilizes and saturates in both GDAL and SGDAL setting. Note that loss curves of ${\mathcal {C}}\circ {\mathcal {F}}$ are maximized owing to our GANs training framework while the loss of conventional setting is minimized, because the yellow curve denotes the classification loss and the blue curves show the discrimination ability. In the meantime, pink curves which show the generator ${\mathcal {G}}$ loss are minimized.

### Analysis and discussion

We estimated and visualized the activation patterns^24^ by using learned spatial filters of the RSTNN-based models for each subject from the III-3a dataset shown in Fig. 4. The topological patterns in the maps coincide with prior neurophysiological findings. Specifically, when a user imagined moving his/her left hand, we observed right-lateralized brain activation patterns, and vice versa with the left hemisphere. Furthermore, imagining foot movements activated the center of a brain, and imagining tongue movements activated the temporal regions. Qualitatively, we observed that the patterns of subject k3b are more prominent than the others. This difference between the patterns of subject k3b and those of the others was related to a difference in their classification performance. In other words, subject k3b’s EEG signals were used more feasibly for learning of class-discriminative features in the network, providing a clearer activation pattern.

Furthermore, we observed that the activation patterns of RSTNN^12^ with adversarial modeling, especially GDAL were more prominent than those of RSTNN with conventional modeling. This result provides insights into the proposed method and its improved ability to learn class-discriminative feature representations from a given dataset. From the results, we observed relatively clearer ERD/ERS patterns (marked by a black arrow) from RSTNN + GDAL and RSTNN + SGDAL estimated patterns as compared to the vanilla RSTNN patterns depicted in Fig. 4.

We further estimated activation patterns with Deep ConvNet and Shallow ConvNet^8^. However, these did not quite reveal neurophysiologically meaningful neural network patterns. We assume that the spatial convolution of layers did not allow for the extraction of spatially meaningful features. Because only one temporal convolutional layer was available before the spatial convolutional layer, thus the networks (Deep ConvNet and Shallow ConvNet^8^) were unable to extract sufficient spectral-temporal information prior to the spatial convolution layer.

## Conclusion

In this study, we described a novel, semi-supervised generative and discriminative adversarial learning framework for BCIs and considered multiple CNN architectures as base generators and discriminator learners. Based on the results of our experiments, the statistically significant improved in performance of the proposed framework validated its effectiveness, especially when a limited number of training samples were provided. We also described how this framework effectively uses unlabeled samples, which facilitate the adaptive updating of network parameters as additional data becomes available (e.g., incremental or lifelong learning paradigms). A visual inspection of the activation pattern maps and comparisons between real and artificial EEG signals in the time and frequency domains allowed us to understand the types of neurophysiological phenomena that were learned by the CNN-based models, their performance improvements and the extent of similarities between the generated signals and real signals. Considering these factors, we conclude that the proposed semi-supervised generative and discriminative adversarial learning framework possesses significant potential for applications in different types of learners and for generation and discrimination in BCI applications.

From a practical standpoint, many challenges remain unaddressed with regard to the use of subject-independent BCIs and reducing the acquisition time of EEG signal. In the present study, all experiments were conducted in a subject-dependent manner. However, for general use, it is important that a BCI system is applicable to any subject. Transfer learning or domain adaptation^16,64^ can be informative for this purpose. Introducing a conditional vector to the proposed framework to generate class-conditioned artificial EEG for data augmentation can also be a possible solution to reduce the acquisition time. Furthermore, this study only exploits artificially generated task-related EEG signals. We believe that there is a possibility of employing unlabeled task-independent EEG signal, e.g., resting-state signals, and their respective artificially generated samples in feature representation learning for EEG analyses and classifications. It would be one of our forthcoming research topics. Finally, even though our work mainly focused on a spontaneous EEG paradigm (i.e., motor imagery), there exist interesting studies^48^ that exploiting artificial EEG samples for BCI applications in evoked EEG paradigms (e.g., SSVEP, P300). Thus, applying our proposed framework to those evoked potentials would also be an intriguing issue.

Additionally, even though this study mainly focused on MI-based BCIs, the proposed method can also be applicable to other types of paradigms (e.g., SSVEP, P300). Thus, applying our proposed framework to other types of EEG-based BCI systems will be interesting.

## Methods

Here, we propose a semi-supervised deep generative adversarial learning framework in Fig. 2, wherein a generator finds a non-linear mapping function from the random noise (i.e., latent space) distribution and the real data distribution, ${\mathbb {P}}_{\mathbf {x}}$, and is therefore capable of generating artificial EEG signals. The discriminator, composed of a feature extractor and a classifier, learns the target-task related EEG feature representations and a class-label mapping function using both real (labeled) and artificial (unlabeled) EEG signals. After training, given a test EEG trial, the discriminator searches the feature extractor and the classifier to identify the user’s intention, which is then converted into a control command to be fed into an external device.

### Adversarial modeling

Despite the availability of advanced approaches for modeling complex data such as EEG signals, data insufficiency for training deep models remains a major concern because generalization requires a huge amount of data. As for BCIs, in general, we have an extremely limited number of samples available for training, e.g., less than one hundred, which are mostly acquired during a calibration session to avoid the potential difficulty in training caused by inter-session variability^16,17^. In this regard, GANs^19^ are emerging as a potential solution to address the aforementioned problem. In GANs, a generator ${\mathcal {G}}(\cdot )$ produces an artificial but realistic samples ${{\tilde{{\mathbf{x}}}}}$ from a random noise vector ${\mathbf {z}}$, i.e., ${{\tilde{\mathbf{x}}}}={\mathcal {G}}({\mathbf {z}}),\ {\mathbf {z}}\sim {\mathbb {P}}_{\mathbf {z}}$. In the same framework, while a generator is trained to synthesize artificial data, a discriminator ${\mathcal {D}}(\cdot )$ is used to discriminate between the artificial and real samples. In this process, the use of the two tunable components, i.e., the generator and discriminator, is akin to playing a minimax game in the framework with no label information involved with the following objective function ${\mathcal {L}}_\text {GANs}({\mathcal {G}},{\mathcal {D}})$^19^:

1 $$\begin{aligned}&\min _{\mathcal {G}}\max _{\mathcal {D}}{\mathcal {L}}_\text {GANs}({\mathcal {G}}({\mathbf {z}}),{\mathcal {D}}({\mathbf {x}})) \end{aligned}$$

2 $$\begin{aligned}{}&{\mathcal {L}}_\text {GANs}={\mathbb {E}}_{{\mathbf {x}}\sim {\mathbb {P}}_{\mathbf {x}}}[\log {\mathcal {D}}({\mathbf {x}})] +{\mathbb {E}}_{{\mathbf {z}}\sim {\mathbb {P}}_{\mathbf {z}}}[\log (1-{\mathcal {D}}({\mathcal {G}}({\mathbf {z}})))] \end{aligned}$$

where ${\mathbb {P}}_{\mathbf {x}}$ and ${\mathbb {P}}_{\mathbf {z}}$ denote, the data distribution and noise distribution, respectively, and ${\mathbb {E}}\left[ \cdot \right]$ refers to an expectation. In this objective function, the parameters of the generator ${\mathcal {G}}$ are trained to fool the discriminator such that ${\mathcal {D}}\left( {\mathcal {G}}({\mathbf {z}})\right) \rightarrow 1$, while those of the discriminator ${\mathcal {D}}$ are trained to distinguish generated artificial data from real data toward ${\mathcal {D}}\left( {\mathcal {G}}({\mathbf {z}})\right) \rightarrow 0$ and ${\mathcal {D}}({\mathbf {x}})\rightarrow 1$.

Notably, the original GANs^19^ are mainly designed and trained in an *unsupervised* manner, and lack learning class-discriminative features. Therefore, to learn class-discriminative feature representations and at the same time, effectively utilize the GANs framework, we modify the discriminator as a feature extractor ${\mathcal {F}}$ combined with a classifier ${\mathcal {C}}$ by adding units to the output layer of the discriminator, such that the additional units can produce target-task related class-label probabilities^59^ as shown in Fig. 2. We refer to this modified framework as a ‘generative and discriminative adversarial learning’ (GDAL) framework. That is, in our GDAL framework, in addition to training the discriminator to distinguish between real and artificial EEG samples, we use it to identify the class labels of real EEG signals. For an *M*-class classification task, we over-parameterize the output layer to have $M+1$ output units. However, in a GDAL framework, the generator still plays the role of mapping a random noise vector to an artificial EEG sample, which is then fed into a discriminator. Furthermore, the discriminator efficiently exploits artificial samples in learning feature representations inherent in task-related EEG signals for class-label identification.

To ensure the effectiveness of the GDAL framework, the original GANs objective function in Eq. (1) also needs to be revised by explicitly denoting the combined feature extractor and classifier ${\mathcal {F}}\circ {\mathcal {C}}(\cdot )$, where ${\mathcal {F}}\circ {\mathcal {C}}(\cdot )={\mathcal {C}}({\mathcal {F}}(\cdot ))$. Thus, the objective function ${\mathcal {L}}_\text {GDAL}({\mathcal {G}},{\mathcal {F}}\circ {\mathcal {C}})$ is defined for the generator, as well as the feature extractor and classifier explicitly combined, as shown below.

3 $$\begin{aligned}&\min _{{\mathcal {G}}}\max _{{\mathcal {F}}\circ {\mathcal {C}}}{\mathcal {L}}_\text {GDAL}({\mathcal {G}}({\mathbf {z}}),{\mathcal {F}}\circ {\mathcal {C}}({\mathbf {x}}_l, {\mathbf {y}}) \end{aligned}$$

4 $$\begin{aligned}{}&{\mathcal {L}}_\text {GDAL}={\mathbb {E}}_{({\mathbf {x}}_l,{\mathbf {y}})\sim {\mathbb {P}}_{({\mathbf {x}}_l,{\mathbf {y}})}}[\log {\mathcal {F}}\circ {\mathcal {C}}({\mathbf {x}}_l,{\mathbf {y}})_{\{1,\cdots ,M\}}]+{\mathbb {E}}_{{\mathbf {z}}\sim {\mathbb {P}}_{\mathbf {z}}}[\log (1-{\mathcal {F}}\circ {\mathcal {C}}({\mathcal {G}}({\mathbf {z}}))_{M+1})] \end{aligned}$$

where ${\mathcal {F}}\circ {\mathcal {C}}\left( \cdot \right) _{a}$ denotes the *a*-th unit in the output layer of the discriminator, and it is assumed that the ($M+1$)-th unit denotes the probability of the sample being real.

### Semi-supervised adversarial modeling

When unlabeled real samples ${\mathbf {x}}_u\sim {\mathbb {P}}_{{\mathbf {x}}_u}$ are available, it is beneficial to use them to boost a model’s robustness and improve generalization by reflecting the characteristics of then additional data distribution pattern^65^. Owing to the unsupervised learning nature of GANs^19^, it is relatively straightforward to utilize unlabeled samples in our model, which we call the ‘Semi-supervised GDAL’ (SGDAL) framework. With the additional unlabeled real samples used during training, there is essentially no change in our framework and model architecture except for the loss function. That is, for unlabeled real samples, a discriminator is required to tune weights connected to a unit whose output indicates the probability of real or artificial cases. Therefore, in a semi-supervised adversarial learning condition, the objective function consists of two parts: one *supervised term* for labeled real EEG samples and the other, an *unsupervised term* for both artificially generated EEG samples and unlabeled real EEG samples. Notably, Eq. (4) deals with both the labeled real samples and the generated samples. Thus, for semi-supervised learning, a modification is needed to account for the unlabeled real samples, for which we further consider a classification loss between the generated and unlabeled real samples in the feature extractor ${\mathcal {F}}$ and the classifier ${\mathcal {C}}$ as follows:

5 $$\begin{aligned}&\min _{\mathcal {G}}\max _{{\mathcal {F}}\circ {\mathcal {C}}} {\mathcal {L}}_\text {SGDAL}({\mathcal {G}}({\mathbf {z}}),{\mathcal {F}}\circ {\mathcal {C}}({\mathbf {x}}, ({\mathbf {y}}))) \end{aligned}$$

6 $$\begin{aligned}{}&{\mathcal {L}}_\text {SGDAL}={\mathcal {L}}_\text {GDAL}+{\mathbb {E}}_{{\mathbf {x}}_u\sim {\mathbb {P}}_{{\mathbf {x}}_u}}[\log {\mathcal {F}}\circ {\mathcal {C}}({\mathbf {x}}_u)_{M+1}] \end{aligned}$$

where ${\mathcal {L}}_\text {GDAL}$ is defined as in Eq. (4).

The SGDAL framework is especially effective when EEG signals are collected gradually over time and/or when the BCI system is used over time. That is, when used in practice, a user induces EEG signals repeatedly, for which we have no ground-truth labels, and these generated signals are useful in updating the network parameters to better reflect a user’s EEG signal patterns. Given this, one noticeable advantage of our SGDAL framework is its inherent way of incremental BCI learning.

Our framework adopts Odena’s work^59^, wherein semi-supervised GANs were applied for computer vision tasks. In this work, we attempt to solve an important application problem in BCI using Odena’s work and devise the training strategies by leveraging recent advanced techniques to stabilize generator and discriminator learning, e.g., Wasserstein distance with gradient penalty to avoid *mode collapse*^53,57^. The following subsection describes organization of those findings.

### Network architectures and learning

Given the physical or mechanical properties of non-invasive EEG, e.g., in the acquisition of signals on the scalp or surface of a brain, it is typically assumed that multi-channel EEG signals provide linear superpositions of the source signals in a volumetric brain^21^. Clearly, multi-channel EEG signals have local and global relationships to one another in both time and space. Hence, to decode a user’s intention, as observed by multi-channel EEG signals, the complex patterns of these is necessary latent signals, in both time and space, must be decoded. We contend that deep networks are capable of disentangling these complicated patterns. However, to design the architectures of the generator ${\mathcal {G}}$ and the combination of the feature extractor and classifier ${\mathcal {F}}\circ {\mathcal {C}}$ in our framework, based on the previous studies^7,8^, we believe that CNNs are good candidates in the case of such complicated patterns.

Given a multi-channel time series input ${\mathbf {x}}\in {\mathbb {R}}^{C\times T}$ with *C* channels and *T* time points, a CNN discovers spatio-temporal relationships by hierarchically interleaving convolution and pooling operations. The convolution operation for EEG representations can be defined in three different ways^7^, depending on the shape of a kernel: 1D temporal (inter-time relations), 1D spatial (inter-channel relations), and 2D spatio-temporal (inter-time and inter-channel joint relationships). For enhancing interpretability, we exploit CNNs with 1D spatial convolutional filters. Additionally, to validate the effectiveness of our SGDAL framework, we employ the existing network architectures available in the literature regarding BCI for designing the feature extractor and the classifier of our study, rather than designing them new. Specifically, we consider the CNN architectures of RSTNN^12^, Deep ConvNet^8^, and Shallow ConvNet^8^.

Given that prior CNNs^8,12^ were primarily designed and trained for classification purposes, the above-mentioned networks are applicable to the use of combined feature extractor and classifier ${\mathcal {F}}\circ {\mathcal {C}}$ in our framework. However, in the case of a generator ${\mathcal {G}}$, a new architecture needs to be designed. Additionally, when training a generator, its stability needs to be ensured. In our work, based on prior study^66^, we regarded the generator as an inverse of the feature extractor and defined its architecture using a deconvolution-like network in which the order of layers in the feature extractor CNN, i.e., RSTNN^12^, Deep ConvNet, and Shallow ConvNet^8^, was reversed, and input was a random noise vector^33,67^. It is empirically validated in designing a generator and a discriminator with an inverse-relationship in their architecture^33^. We also used a bilinear-resize up-sampling technique to deconvolute operations, rather than zero-inserting^34^ to enhance the quality of generated EEG signals.

To avoid a potential mode collapse^67^ during training, we used *Wasserstein divergence* with a gradient penalty^53^ and feature matching techniques^66^ by modifying the objective function in Eq. (4) and Eq. (6) as follows:

7 $$\begin{aligned} {\mathcal {L}}_\text {GDAL}= & {} {\mathbb {E}}_{({\mathbf {x}}_l,{\mathbf {y}})\sim {\mathbb {P}}_{({\mathbf {x}}_l,{\mathbf {y}})}}[\log {\mathcal {F}}\circ {\mathcal {C}}({\mathbf {x}}_l,{\mathbf {y}})_{\{1,\cdots ,M\}}]+{\mathbb {E}}_{{\mathbf {z}}\sim {\mathbb {P}}_{\mathbf {z}}}[{\mathcal {F}}({\mathcal {G}}({\mathbf {z}}))]+\lambda {\mathbb {E}}_{{\hat{\mathbf {x}}}\sim {\mathbb {P}}_{\hat{\mathbf {x}}}}[(||\nabla _{{\hat{\mathbf {x}}}}{\mathcal {F}}({\hat{\mathbf {x}}} )||_2-1)^2] \end{aligned}$$

8 $$\begin{aligned} {\mathcal {L}}_\text {SGDAL}= & {} {\mathcal {L}}_\text {GDAL} + {\mathbb {E}}_{{\mathbf {x}}_u\sim {\mathbb {P}}_{{\mathbf {x}}_u}}[{\mathcal {F}}({\mathbf {x}}_u)] \end{aligned}$$

where ${\mathcal {F}}$ is a feature extractor in a discriminator, i.e., a subnetwork before the output layer, $\lambda$ is a hyperparameter, $\hat{{\mathbf {x}}}=\epsilon {\mathbf {x}} + \left( 1-\epsilon \right) {\mathcal {G}}\left( {\mathbf {z}}\right)$, and $\epsilon \in {\mathbb {R}}$ is a random number between 0 and 1. Algorithm 1 describes the pseudo-codes for learning with the objective functions defined above.

During testing, given a new EEG signal ${\mathbf {x}}_{\text {new}}$, we use the combination of the feature extractor and classifier ${\mathcal {F}}\circ {\mathcal {C}}$ from our adversarial models, i.e., GDAL and SGDAL, with output units related to the class labels, ignoring the $(M+1)$-th unit related to a real/artificial decision). That is, the decision function is defined as

9 $$\begin{aligned} \hat{k} = {\mathop {{{\,\mathrm{argmax}\,}}}\limits _{k}} {\mathcal {F}}\circ {\mathcal {C}}\left( {\mathbf {x}}_{\text {new}}\right) _{\{1,\dots ,k,\dots ,M\}}. \end{aligned}$$

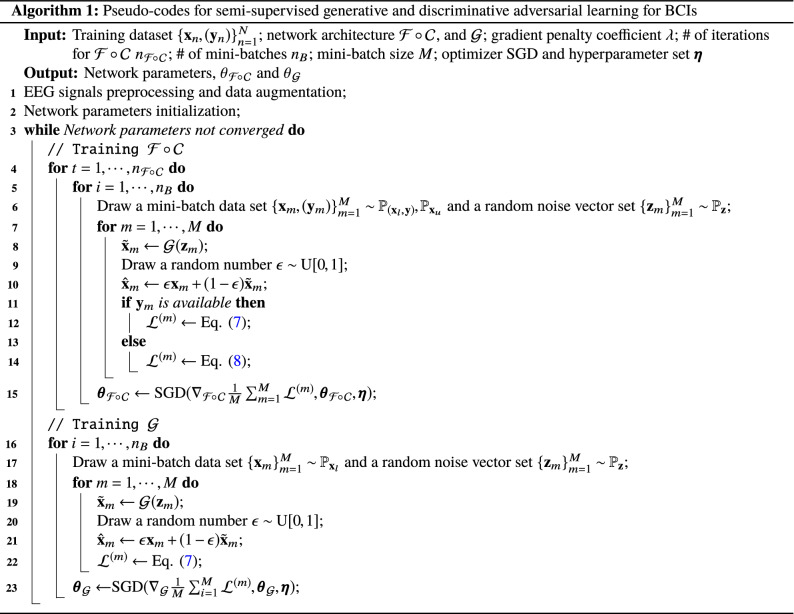

### Investigating the learned network weights

Owing to the advancements and achievements of deep learning, researchers have been paying more attention to the interpretation of trained models. However, interpretation of learned kernel weights in a CNN is still a challenge as the inter-mixed non-linear operations as progressing towards the output layer of a network. Recent studies of^8,10,23^ devised ways to understand the learned features representations or network weights. Schirrmeister et al.^8^ conducted visual analysis by calculating correlations of (input)-(unit responses)-(outputs). Lawhern et al.^10^ presented three different approaches, namely, summarization of unit responses, visualization of kernel weights, and calculation of gradient-based single-trial feature relevance. Sturm et al.^23^ introduced a layer-wise relevance propagation to identify which components in an input influenced the final output. Concisely, the aforementioned previous studies analyzed network responses or visualized convolution weights, which correspond to spatial filters. However, to the best of our understanding, such a method is good to identify the observation that affected the final decision. However, it does not explicitly describe the underlying patterns, which are helpful (1) to understand neurophysiological insights shared across samples/subjects and (2) to identify discriminative characteristics the trained network commonly exploits for classification.

Therefore, we introduce a method for investigating learned network parameters making them neurophysiologically plausible and visualizing them using topographic maps. Earlier, Haufe et al.^24^ proposed a method for the interpretation of weight vectors in multivariate neuroimaging, called an *activation pattern*, which is based on a forward-backward modeling concept. Essentially, the classification or decoding task used in the framework proposed here may also be regarded as a backward process by which a user’s intention is inferred from the fundamental induction of observed EEG signals. That is, our discriminative model extracts features from an input sample by applying filtering operations in a non-linear manner. These features are then used for classification rather than showing how observed EEG signals are evoked or activated by a user’s intention. Thus, for a concrete and intuitive understanding of learned network parameters, it is necessary to revert to a forward process computational model. With reference to Haufe et al.’s work^24^, we derive the following equation (for the proof, refer to the original Haufe et al.’s work^24^) to estimate unknown activation patterns from learned weight parameters:

10 $$\begin{aligned} {\mathbf {A}}\equiv {{\varvec{\Sigma }}}_{\text {input}}{\mathbf {W}}{{\varvec{\Sigma }}}_{\text {output}}^{-1} \end{aligned}$$

where ${\mathbf {A}}$ denotes a set of activation patterns, each of which corresponds to learned weights ${\mathbf {W}}$, and ${{\varvec{\Sigma }}}_{\text {input}}$ and ${{\varvec{\Sigma }}}_{\text {output}}$ denote, respectively, the covariance matrices of the input vector and corresponding output from the learned layer. In particular, by mapping the activation patterns, ${\mathbf {A}}$, estimated with the weights of spatial convolution kernels in the form of a topography, which we refer to as an ‘activation pattern map,’ we visualize latent activations and obtain insights into the neurophysiological characteristics of target tasks.

## Data Availability

We used three publicly available datasets.

## Code availability

All codes used in our experiments are available at http://deepbci.korea.ac.kr/opensource/opensw/.
